# TET3‐Mediated m5C Modification of CCAT2 Accelerates Cardiac Microvascular Endothelial Cell Damage in Acute Coronary Syndrome

**DOI:** 10.1002/kjm2.70128

**Published:** 2025-11-29

**Authors:** Jun‐Cheng Liu, Wen‐Juan Wang, Ting‐Ting Zhang, Qi‐Chong Yang, Haliminai Dilimulati, Song‐Tao An

**Affiliations:** ^1^ Department of Cardiology Henan Province People's Hospital, People's Hospital of Zhengzhou University Zhengzhou China; ^2^ National Health Commission Key Laboratory of Cardiovascular Regenerative Medicine, Central China Subcenter of National Center for Cardiovascular Diseases, Henan Cardiovascular Disease Center Fuwai Central‐China Cardiovascular Hospital, Central China Fuwai Hospital of Zhengzhou University Zhengzhou China; ^3^ Henan Provincial Cell and Gene Engineering Technology Research Center for Cardiovascular Disease, Fuwai Central‐China Cardiovascular Hospital Zhengzhou China; ^4^ Center for Clinical Single‐Cell Biomedicine Henan Province People's Hospital Zhengzhou China

**Keywords:** CCAT2, coronary heart disease, FUS, m5C, TET3

## Abstract

Acute coronary syndrome (ACS) is a clinical syndrome involving myocardial ischemia. This study aimed to elucidate the mechanism of TET3 in ACS‐induced CMEC damage, thereby identifying a new target for ACS treatment. The expression of TET3 in ACS patients and healthy subjects was analyzed. CMECs were stimulated with ox‐LDL and transfected with si‐TET3 for the detection of TET3 RNA and protein levels. Cell proliferation, apoptosis, and angiogenesis were evaluated. Subsequently, m5C modification and TET3 enrichment on CCAT2 were assessed, and CCAT2 stability was measured. The binding relationships between CCAT2 and FUS and between FUS and TRIM14 mRNA were analyzed. Additionally, lncRNA CCAT2 inhibition or TRIM14 overexpression in combination with si‐TET3 treatment was conducted to verify the underlying mechanism. TET3 was strongly expressed in serum from ACS patients and ox‐LDL‐stimulated CMECs, and silencing TET3 reduced ox‐LDL‐induced CMEC damage. TET3 removed m5C modification on CCAT2 to decrease CCAT2 stability and expression. With TRIM14, CCAT2 competes to bind to FUS to suppress TRIM14 expression. CCAT2 knockdown or TRIM14 overexpression partially reversed the protective effect of si‐TET3 on CMEC damage. In conclusion, TET3 removed m5C modification to inhibit CCAT2 expression and reduced the binding relationship between CCAT2 and FUS to upregulate TRIM14, thereby exacerbating CMEC damage in ACS.

## 1 Introduction

Coronary heart disease (CHD) is related to a series of consequences, including serious angina, coronary dysfunction and remodeling, and myocardial infarction, that can result in high incidence and death rates, and its pathogenesis is triggered by senility, family history, cigarette consumption, and unhealthy lifestyles [1]. Acute coronary syndrome (ACS) is the acute critical phase of CHD, caused by thrombosis induced by plaque rupture, erosion, or the formation of calcified nodules, which leads to acute myocardial ischemia and hypoxia and clinically includes ST‐segment elevation myocardial infarction (STEMI), non‐ST‐segment elevation myocardial infarction (NSTEMI), and unstable angina [2]. Current treatment approaches for ACS include pharmacological therapy (such as antiplatelet drugs and statins), percutaneous coronary intervention, and coronary artery bypass grafting [1]. Nevertheless, although protocols for the detection and prevention of CHD have been intensively published, insufficient awareness, ineffective monitoring, and inadequate clinical treatment could still expedite the spread of CHD [3]. Functionally, cardiac microvascular endothelial cells (CMECs) participate in the biological processes of ACS, and CMEC damage further facilitates myocardial ischemic injury and heart failure [4]. Thus, targeting CMEC damage is an intriguing prospect for developing ACS prevention protocols.

Ten‐eleven translocation family proteins (TETs) modulate cellular development, gene expression, transcriptional modification, and microenvironmental homeostasis during various pathological events through 5‐methylcytosine (m5C) oxidation to alter DNA methylation [5]. TETs control DNA methylation and m5C modification to alter downstream gene expression to mediate disease progression [6]. TET3 expression is activated in coronary artery disease (CAD), and it accelerates CAD initiation and severity by inducing the migratory and invasive abilities of vascular smooth muscle cells [7]. TET3 coordinates DNA methylation by oxidizing m5C to regulate long noncoding RNA (lncRNA) expression in a wide range of disorders [5]. Nevertheless, few studies have reported the role of TET3‐mediated m5C modification on lncRNAs in ACS. LncRNAs are differentially expressed in ACS, which is related to inflammatory infiltration, perivascular adipose tissue, and atherosclerosis [8]. The lncRNA colon cancer‐associated transcript 2 (CCAT2) exerts a protective effect on myocardial ischemia/reperfusion (MI/R), as it can reduce oxidative damage, restrict cardiomyocyte apoptosis, and restore heart function [9]. CCAT2 participates in the competitive endogenous RNA (ceRNA) network to regulate its downstream targets to mediate neoplasm occurrence, aggressiveness, and prognosis [10]. Moreover, lncRNAs can participate in biological functional changes by directly interacting with RNA‐binding proteins [11], but the RNA–protein interaction mechanism of CCAT2 in ACS has rarely been studied. In our study, it was predicted through an RNA–protein interaction prediction website that CCAT2 could competitively bind to FUS RNA binding protein (FUS) with tripartite motif‐containing 14 (TRIM14). TRIM14 acts as a hub factor in ceRNA crosstalk to alter neoplasm progression [12]. TRIM14 is a detrimental cytokine in heart diseases, as it accelerates cardiomyocyte dysfunction and fibrosis, inflammatory reactions, and ischemia/reperfusion damage [13]. TRIM14 promotes ox‐LDL‐induced CMEC damage and dysfunction in CAD by sponging related genes [14]. As an RNA‐binding protein, FUS can interact with upstream lncRNAs to mediate the inflammatory response, cellular deficiency, and cardiac dysfunction [15]. Overall, we hypothesize that TET3 might be a detrimental factor in ACS alleviation, and functional assays were performed to elucidate the mechanism of TET3 in ACS‐induced CMEC damage via manipulation of the CCAT2/TRIM14/FUS axis, thus broadening the possibility of an ACS regimen.

## 2 Materials and Methods

### 2.1 Ethics Statement

This study was approved by the Clinical Ethical Committee of our hospital. Informed consent was obtained from all subjects. Every step was rigorously performed in accordance with the *Declaration of Helsinki*.

### 2.2 Research Subjects

From February 2022 to February 2024, 142 ACS patients who underwent coronary angiography or coronary computed tomography at People's Hospital of Zhengzhou University were included in our study (NSTE‐ACS accounted for 76% and STEMI for 24%, with 80 males and 62 females, aged 40 to 79 years, with an average age of 61.29 ± 8.94 years). All the enrolled patients met the 2023 ESC Guidelines for the Management of Acute Coronary Syndromes [16]. The recruitment conditions were as follows: (1) typical chest pain symptoms plus coronary stenosis > 50% and (2) absence of other serious cardiovascular diseases, acute or chronic infection, severe liver and kidney failure, tumors or autoimmune disorders, major surgery/trauma within the past 3 months, or a history of immunosuppressant use. A total of 120 healthy subjects were included in this research (71 males and 59 females aged 40–79 years, with an average age of 60.31 ± 8.44 years). General information for both groups is shown in Table 1.

**TABLE 1:** General comparisons between the ACS and the control groups.

Features	Control group (*n* = 120)	ACS group (*n* = 142)	*p*
Age	60.31 ± 8.44	61.29 ± 8.94	0.365
Gender (male, %)	71 (59.17%)	80 (56.34%)	0.644
BMI (kg/m^2^)	23.61 ± 1.57	24.02 ± 2.09	0.081
Smoking history (*n*, %)	43 (35.83%)	62 (43.66%)	0.198
Alcohol history (*n*, %)	32 (26.67%)	47 (33.10%)	0.258
FPG (mmol/L)	5.35 ± 0.89	5.51 ± 1.22	0.478
TC (mmol/L)	4.10 ± 0.58	4.34 ± 0.87	0.057
TG (mmol/L)	1.40 ± 0.41	1.53 ± 0.47	0.032
LDL‐C (mmol/L)	2.14 ± 0.78	2.36 ± 0.88	0.035
HDL‐C (mmol/L)	1.28 ± 0.28	1.18 ± 0.26	0.002
CK‐MB (μg/L)	1.69 ± 0.34	3.23 ± 1.28	< 0.001
cTnI (ng/mL)	0.04 ± 0.02	0.25 ± 0.08	< 0.001

*Note*: Quantitative data were expressed as mean ± standard deviation and analyzed using *t* test and Mann–Whitney U test. Counting data were presented as *n* (%) and analyzed using chi‐square test.

Abbreviations: ACS, acute coronary syndrome; BMI, body mass index; CK‐MB, creatine kinase‐myocardial band; cTnI, cardiac troponin I; FPG, fasting plasma glucose; HDL‐C, high‐density lipoprotein cholesterol; LDL‐C, low‐density lipoprotein cholesterol; TC, total cholesterol; TG, triglyceride.

### 2.3 Sample Collection and Analysis

Before coronary angiography, 10 mL of fasting peripheral blood was collected from all the enrolled subjects in the early morning. The blood samples were centrifuged at 2500 × *g* for 20 min at 4°C to isolate the serum, which was preserved at −80°C until further use. Blood glucose and blood lipids, including serum total cholesterol, triglycerides, low‐density lipoprotein cholesterol (LDL‐C), high‐density lipoprotein cholesterol (HDL‐C), creatine kinase isoenzyme (CK‐MB), troponin I (cTnI), and fasting blood glucose, were measured with an automatic biochemical analyzer (AU5800, Beckman Coulter Inc., Brea, CA, USA). Serum TET3 expression and CCAT2 levels were analyzed by reverse transcription quantitative polymerase chain reaction (RT–qPCR) and western blotting.

### 2.4 Cell Culture and Treatment

Human CMECs (Procell Life Science & Technology Co. Ltd., Wuhan, Hubei, China) were incubated in Dulbecco's modified Eagle's medium (Gibco, BRL, CA, USA) supplemented with 10% fetal bovine serum (FBS; HyClone, Carlsbad, CA, USA) with 5% CO_2_ at 37°C under humidified conditions. After reaching 80% confluence, the passage three CMECs were incubated with oxidized (ox)‐LDL at different concentrations (0, 50, 100, or 150 μg/mL; Solarbio Science & Technology Co. Ltd., Beijing, China) for 24 h to induce the CMEC injury model. CMECs were assigned to the control group (the CON group, untreated CMECs), the ox‐LDL group (injured CMECs without treatment), the small interfering‐negative control group (the si‐NC group, injured CMECs treated with negative control siRNA), the TET3 siRNA treatment group (the si‐TET3 group, injured CMECs treated with TET3 siRNA plasmids), the CCAT2 siRNA group (the si‐CCAT2 group, injured CMECs treated with CCAT2 siRNA plasmids), the empty plasmid group (the oe‐NC group, injured CMECs treated with empty plasmids), the overexpression TRIM14 group (the oe‐TRIM14 group, injured CMECs transfected with the oe‐TRIM14 plasmid), and the FUS siRNA group (the si‐FUS group, injured CMECs transfected with FUS siRNA plasmids). All siRNAs and plasmids (GenePharma Co. Ltd., Shanghai, China) were transfected into CMECs following the instructions of Lipofectamine 3000 transfection reagent (Thermo Fisher Scientific, Rockford, IL, USA). After 48 h of transfection, the following experiments were carried out.

### 2.5 RT–qPCR

TRIzol reagent (Invitrogen, Carlsbad, CA, USA) was used to isolate total RNA from serum or CMECs. Afterwards, the RNA was reverse transcribed into complementary DNA with a PrimeScript RT Kit (Takara, Dalian, Liaoning, China). RT–qPCR was carried out using SYBR Green Master Mix (Roche, Shanghai, China) on a CFX97 system (Bio‐Rad Laboratories, Hercules, CA, USA) following a standardized protocol. The RT–qPCR amplification conditions were as follows: 42°C for 5 min; 95°C for 10 s; and 40 cycles of 95°C for 5 s, 60°C for 20 s, and 72°C for 15 s, with glyceraldehyde‐3‐phosphate dehydrogenase (GAPDH) as the internal reference. Relative gene expression was verified with the 2^−ΔΔCt^ method. The primer sequences are shown in Table 2.

**TABLE 2:** Primers sequence of RT‐qPCR.

Gene	Forward (5′‐3′)	Reverse (5′‐3′)
CCAT2	TGGACTGGAAGTCAAGAGCC	CCCAGATGCAGAGAACGAGG
FUS	ATGGCCTCAAACGATTATACCCA	GTAACTCTGCTGTCCGTAGGG
TRIM14	TACATTACAGACGCCATTGGAC	GGGCTGGTTTTCAACAAGGT
GAPDH	GTCTCCTCTGACTTCAACAGCG	ACCACCCTGTTGCTGTAG CCAA

Abbreviations: CCAT2, colon cancer‐associated transcript 2; FUS, fused in sarcoma; GAPDH, glyceraldehyde‐3‐phosphate dehydrogenase; RT‐qPCR, reverse transcription‐quantitative polymerase chain reaction; TRIM14, tripartite motif‐containing 14.

### 2.6 Western Blot Analysis

CMECs were collected, and cell extracts were lysed using radioimmunoprecipitation assay cell lysis buffer (Beyotime, Shanghai, China). The protein concentration was analyzed by a bicinchoninic acid protein assay kit (Beyotime). The proteins were subsequently separated by sodium dodecyl sulfate (SDS)‐polyacrylamide gel electrophoresis and then transferred onto nitrocellulose membranes, which were blocked with 5% FBS for 1 h. The membranes were incubated with primary antibodies against TET3 (ab231785; 1:1000; Abcam, Cambridge, UK), FUS (ab124923; 1:1000; Abcam), TRIM14 (15742‐1‐AP; 1:500; Proteintech, Wuhan, Hubei, China), and β‐actin (ab8227; 1:1000; Abcam) at 4°C overnight and then rinsed three times with tris‐buffered saline supplemented with Tween‐20. The membranes were incubated with a secondary antibody (ab97051; 1:5000; Abcam) for 2 h. An enhanced chemiluminescence protein blot assay system (GE Healthcare, Amersham, UK) was used to visualize the protein bands, and the gray values were calculated using NIH ImageJ software (National Institutes of Health, Bethesda, MD, USA).

### 2.7 Cell Counting Kit‐8 (CCK‐8) Method

Cell proliferation was assessed by a CCK‐8 kit (Beyotime). CMECs (5 × 10^3^ cells/well) from each group were cultured in 96‐well plates, which were subsequently incubated with CCK‐8 solution (10 μL/well) for 2 h. The optical density at a wavelength of 450 nm was determined by a microplate reader (Bio‐Rad).

### 2.8 5‐Ethynyl‐2′‐Deoxyuridine (EdU) Assay

The cellular DNA replication capacity was evaluated using an EdU assay kit (RiboBio Biotechnology Co. Ltd., Guangzhou, Guangdong, China). After routine treatment, CMECs (1 × 10^4^ cells/well) were seeded on 96‐well plates, with three parallel wells in each group. CMECs were cultured with 50 μM EdU (100 μL) for 2 h, fixed with 4% paraformaldehyde for 20 min, cultured with 2% glycine for 15 min, and permeabilized with 0.5% Triton X‐100. CMECs were subsequently stained with Apollo solution for 30 min in the dark, cultured with 4′,6‐diamidino‐2‐phenylindole in the dark for 10 min, and rinsed 3 times with 0.5% Triton X‐100. CMECs were then observed using a fluorescence microscope (Olympus FSX100, Tokyo, Japan), with five random visual fields selected. The blue fluorescence represents CMECs, whereas the red fluorescence represents EdU‐positive replicating cells. The percentage of EdU‐positive cells was determined as the ratio of red‐fluorescent cells to total blue‐fluorescent cells.

### 2.9 Matrix Tube Formation Assay

The ability of CMECs to construct vascular rings in vitro was evaluated through a matrix tube formation assay. In short, CMECs from each transfection group were seeded into 96‐well plates covered with matrix (BD Biosciences, San Jose, CA, USA). After 48 h of culture, the CMECs were observed under a microscope (Leica, Mannheim, Germany) for the formation of tubes, and photos were taken. The length of each tube was measured using ImageJ software with the angiogenesis analyzer plugin.

### 2.10 Flow Cytometry

Apoptosis was measured using propidium iodide (PI)/fluorescein isothiocyanate (FITC)‐annexin V staining (BD Pharmingen, San Diego, CA, USA). CMECs were rinsed with phosphate‐buffered saline 3 times, stained with PI/FITC‐annexin V using 50 μg/mL RNase A (Sigma–Aldrich Inc., St. Louis, MO, USA), and cultured in the dark for 1 h. Apoptotic and necrotic cells were classified using FACS (Beckman Coulter, Fullerton, CA, USA), and the data were analyzed using FlowJo software (Tree Star software, San Carlos, CA, USA). The standards for apoptotic cells were as follows: the upper left quadrant (Q1) represents mechanically damaged cells (FITC−/PI+), the upper right quadrant (Q2) represents late apoptotic or necrotic cells (FITC+/PI+), the lower right quadrant (Q3) represents early apoptotic cells (FITC+/PI−), and the lower left quadrant (Q4) represents healthy viable cells (FITC−/PI−). The apoptosis rate = Q2 + Q3. Three independent experiments were performed.

### 2.11 Methylated RNA Immunoprecipitation (MeRIP)‐qPCR

MeRIP assays were carried out with a Magna MeRIP mRNA methylation kit (Millipore Corp., Billerica, MA, USA) to analyze m5C levels in CCAT2. Briefly, total RNA was separated from CMECs by TRIzol (Invitrogen). Subsequently, 5 μg of RNA was converted into a fragment at a nucleoside length of approximately 200, which was then cultured with a mixture of magna protein G beads and m5C antibody (ab10805; Abcam) at 4°C overnight. After immunoprecipitation of the RNA–antibody complexes, RNA was purified from the beads. In addition, the number of enriched RNA fragments containing m5C modifications was determined by RT–qPCR.

### 2.12 RNA Stability Assay

CMECs were treated with the RNA synthesis inhibitor actinomycin D (ACT‐D, 5 μg/mL), and RNA was isolated using TRIzol reagent at various time points (0, 6, 12, 18, and 24 h) after treatment. The extracted RNA was reverse transcribed into complementary DNA with an iScript cDNA synthesis kit (Bio‐Rad). Additionally, the half‐lives of CCAT2 and TRIM14 were determined using SYBR Green Supermix (Bio‐Rad) through RT–qPCR, with GAPDH used as a standard control.

### 2.13 RNA Immunoprecipitation (RIP) Assay

RIP was carried out with an EZ Magna RIP RNA binding protein IP kit (Millipore). CMECs were lysed with RIP lysis buffer before the cell lysate was incubated with RIP buffer coupled with anti‐FUS antibody (#4885; Cell Signaling Technology, Danvers, MA, USA) and anti‐immunoglobulin G (IgG; #2729; Cell Signaling Technology) at 4°C for 6 h. The magnetic beads were rinsed with buffer solution, and the complexes were treated with 0.1% SDS and 0.5 mg/mL proteinase K at 55°C for 30 min to digest the protein. The immunoprecipitated RNA was purified, and the enrichment of CCAT2 and TRIM14 in the immunoprecipitated RNA was examined by RT–qPCR.

### 2.14 RNA Pull‐Down Assay

CCAT2 or TRIM14 was transcribed with T7 RNA polymerase (Roche) and purified with an RNeasy Plus Mini Kit (QIAGEN, Germantown, MD, USA), and the mixture was labeled with biotin RNA (Roche). Biotinylated RNA was subsequently cultured with CMEC lysate supplemented with streptavidin‐conjugated magnetic beads (Invitrogen). Eluted protein was examined by western blot analysis.

### 2.15 Bioinformatics Analysis

The nuclear localization of CCAT2 was predicted through the lncLocator database (http://www.csbio.sjtu.edu.cn/bioinf/lncLocator/) [17]. m5C modification of CCAT2 was predicted through the iRNA‐m5C website (http://lin‐group.cn/server/iRNA‐m5C/service.html) [18], and RNA–protein interactions were predicted through the RPISeq database (http://pridb.gdcb.iastate.edu/RPISeq/index.html) [19].

### 2.16 Statistical Analysis

Prism v8.0 software (GraphPad Software Inc., La Jolla, CA, USA) was used to analyze the data; the data are expressed as *n* (%), and the chi‐square test was used for analysis. Our results were examined with respect to normality distribution and homogeneity by the Kolmogorov–Smirnov test, and the data are presented as the mean ± standard deviation. Comparisons between two groups were conducted with *t* tests or Mann–Whitney U tests, and comparisons among various groups were performed with one‐way or two‐way analysis of variance (ANOVA). Tukey's multiple comparisons test was used for post hoc tests. ROC curve analysis was performed to analyze the diagnostic value of serum TET3 protein levels in patients with ACS, and Pearson's correlation coefficient was used for correlation analysis. The *p* value was obtained by a two‐tailed test, with *p* < 0.05 indicating statistical significance.

## 3 Results

### 3.1 TET3 Is Strongly Expressed in Serum From ACS Patients and ox‐LDL‐Stimulated CMECs

**FIGURE 1:**
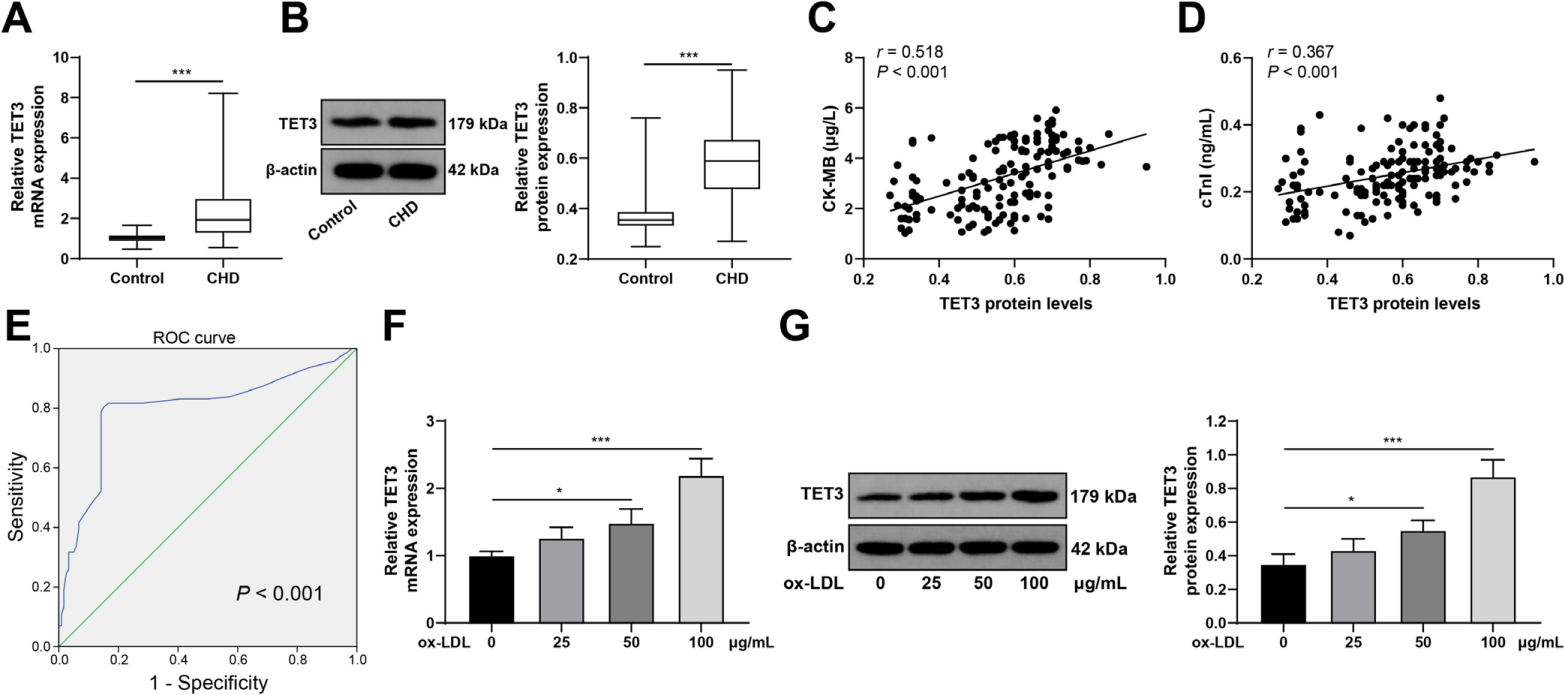
TET3 is strongly expressed in serum from ACS patients and ox‐LDL‐stimulated CMECs. (A and B) Serum TET3 mRNA and protein levels in ACS (*n* = 142) and healthy (*n* = 120) subjects were detected by RT–qPCR (A) and western blot analysis (B), respectively. (C and D) Relationships between serum TET3 protein levels in ACS patients and the levels of CK‐MB (C) and cTnI (D) were analyzed by Pearson's correlation coefficient. (E) ROC curve of serum TET3 protein levels in patients with ACS. (F and G) TET3 mRNA and protein levels in response to different concentrations of ox‐LDL (0, 25, 50, and 100 μg/mL) were verified by RT–qPCR (F) and western blot analysis (G). Three independent operations were conducted. The data are presented as the mean ± standard deviation. The Mann–Whitney U test was used for data comparisons between two groups in Panels A and B, and one‐way ANOVA was used for data comparisons among multiple groups in Panels F and G. Tukey's multiple comparisons test was used for post hoc tests. *p < 0.05, **p < 0.01, ***p < 0.001.

TET3 participates in the initiation of CAD [7], but the understanding of how it affects ACS remains elusive. Here, TET3 expression in serum from ACS patients was measured via RT–PCR and western blot analysis, which revealed that the ACS group exhibited upregulated TET3 compared with the control group (both *p* < 0.01; Figure 1A,B). Pearson's correlation analysis revealed an association between serum TET3 protein levels and myocardial damage indicators in ACS patients, and the findings revealed that serum TET3 protein levels were positively related to CK‐MB and cTnI levels (all *r* > 0, *p* < 0.001; Figure 1C–D). ROC curve analysis (Figure 1E) revealed that the serum TET3 protein level predicted a ROC area under the curve for ACS of 0.798, and the 95% CI was 0.741–0.855; the sensitivity was 80.30%, and the specificity was 85.00%, indicating that serum TET3 protein was highly expressed in ACS and offered considerable diagnostic value for ACS. In addition, TET3 expression in CMECs treated with different concentrations of ox‐LDL (0, 25, 50, and 100 μg/mL) was determined by RT–qPCR and western blot analysis. TET3 expression was positively related to the ox‐LDL concentration, with significant upregulation of TET3 observed at 50 and 100 μg/mL ox‐LDL (*p* < 0.05; Figure 1F–G).

### 3.2 TET3 Silencing Alleviates Ox‐LDL‐Induced CMEC Damage

**FIGURE 2:**
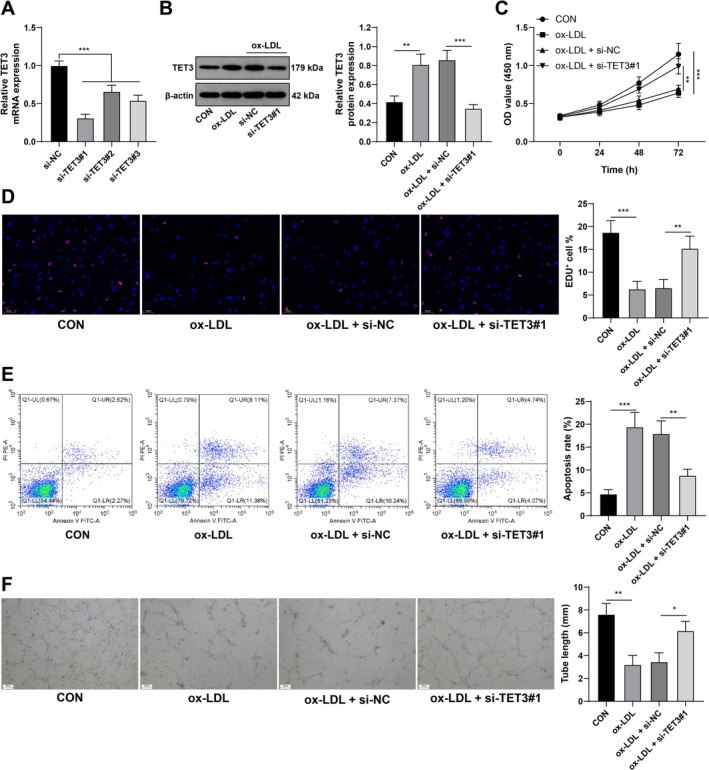
TET3 silencing alleviates ox‐LDL‐induced CMEC damage. (A) TET3 mRNA expression in CMECs transfected with si‐NC and si‐TET3 (#1, #2, and #3) for 48 h was detected by RT–qPCR. (B) TET3 protein levels in CMECs transfected with si‐TET3 and treated with 100 μg/mL ox‐LDL for 24 h were determined by western blot analysis. (C) CMEC proliferation was verified by the CCK‐8 method. (D) The DNA replication capacity of CMECs was evaluated by an EdU assay. (E) CMEC apoptosis was evaluated by flow cytometry. (F) The ability of CMECs to form vascular rings in vitro was measured through a matrix tube formation assay. Three independent operations were conducted. The data are presented as the mean ± standard deviation. One‐way ANOVA was used to compare the data among multiple groups in Panels A, B, D, and F, and two‐way ANOVA was used to compare the data among multiple groups in Panel C. Tukey's multiple comparisons test was used for post hoc tests. *p < 0.05, **p < 0.01, ***p < 0.001.

To elucidate the mechanism of TET3 in ox‐LDL‐induced CMEC damage, CMECs were transfected with si‐TET3 for 48 h to inactivate TET3 expression (all *p* < 0.01; Figure 2A). si‐TET3#1 was selected for subsequent transfection because it resulted in the most effective downregulation efficiency, after which 100 μg/mL ox‐LDL was used to treat CMECs for 24 h (both *p* < 0.01; Figure 2B). Our data revealed that TET3 silencing reduced the suppressive effect of ox‐LDL on CMEC proliferation (all *p* < 0.01; Figure 2C,D), suppressed apoptosis (all *p* < 0.01; Figure 2E), and promoted angiogenesis (all *p* < 0.01; Figure 2F), demonstrating that TET3 silencing alleviated ox‐LDL‐induced CMEC damage.

### 3.3 TET3 Removes m5C Modification on CCAT2 to Reduce Its Stability

**FIGURE 3:**
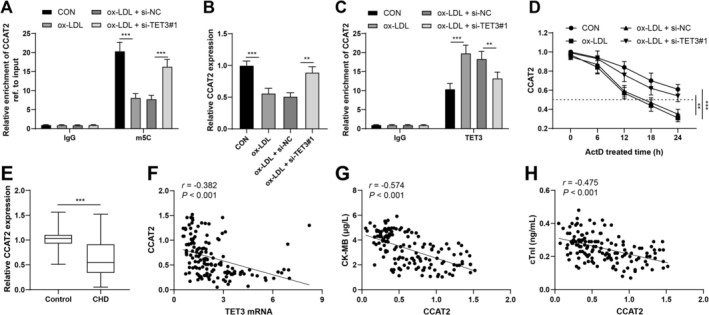
TET3 removes m5C modification on CCAT2 to reduce its stability. (A) The m5C methylation level of CCAT2 upon transfection with si‐TET3 was verified by MeRIP‐qPCR. (B) CCAT2 expression upon si‐TET3 transfection was determined by RT–qPCR. (C) CMEC lysis solution was collected, RIP was carried out using a TET3 antibody, and compared with the IgG group, the TET3 group showed enhanced CCAT2 enrichment. (D) CMECs transfected with control or si‐TET3 were exposed to ACT‐D (5 μg/mL), cellular RNA was separated at designated time points, and the effect of si‐TET3 on the half‐life of CCAT2 was evaluated by RT–qPCR. (E) Serum CCAT2 expression in the healthy control group (*n* = 120) and the ACS group was verified by RT–qPCR. (F–H), Relationships of serum CCAT2 expression in ACS patients with TET3 mRNA (F), CK‐MB (G), and cTnI (H) levels were analyzed by Pearson's correlation coefficient. Three independent operations were conducted. The data are presented as the mean ± standard deviation. The Mann–Whitney U test was used to compare the data between two groups in Panel E; one‐way ANOVA was used to compare the data among multiple groups in Panel B; and two‐way ANOVA was used to compare the data among multiple groups in Panels A, C, and D. Tukey's multiple comparisons test was used for post hoc tests. **p < 0.01, ***p < 0.001.

TET3 can function as a DNA dioxygenase to accelerate m5C modification to 5‐hydroxymethylcytosine (5hmC) on DNA [6], but how TET3‐mediated RNA m5C modification affects ACS‐induced microvascular endothelial cell damage is unclear. CCAT2 is poorly expressed in MI/R cells [9], and CCAT2 is localized mainly in the nucleus. In addition, the iRNA‐m5C website predicted m5C modification sites on CCAT2. To determine how m5C modification modulates CCAT2 expression, si‐TET3 was transfected into CMECs, while ox‐LDL was used to treat CMECs. The results revealed that ox‐LDL downregulated CCAT2 and decreased m5C levels on CCAT2 and that TET3 silencing promoted m5C levels on CCAT2 in CMECs (all *p* < 0.001; Figure 3A) and activated CCAT2 expression (all *p* < 0.01; Figure 3B). RIP assays demonstrated that compared with IgG, TET3 antibody‐enriched CCAT2 and ox‐LDL treatment further increased CCAT2 enrichment in CMECs, which was reduced by si‐TET3 (all *p* < 0.01; Figure 3C). ACT‐D determination was performed to assess whether TET3 could manipulate CCAT2 stability to regulate its expression, and the results revealed that the ox‐LDL group had a shortened CCAT2 half‐life in CMECs, which was reversed in the si‐TET3 group (*p* < 0.01; Figure 3D). RT–qPCR revealed that compared with the healthy group, the ACS group exhibited downregulated CCAT2 (*p* < 0.001; Figure 3E). Pearson's correlation analysis revealed that CCAT2 expression in ACS serum was negatively related to the levels of TET3 mRNA, CK‐MB, and cTnI (all *r* < 0, *p* < 0.001; Figure 3F–H). Collectively, TET3 removed m5C modification on CCAT2 and reduced CCAT2 stability to downregulate it.

### 3.4 CCAT2 Competes With TRIM14 mRNA for Binding to FUS, Thereby Downregulating TRIM14

**FIGURE 4:**
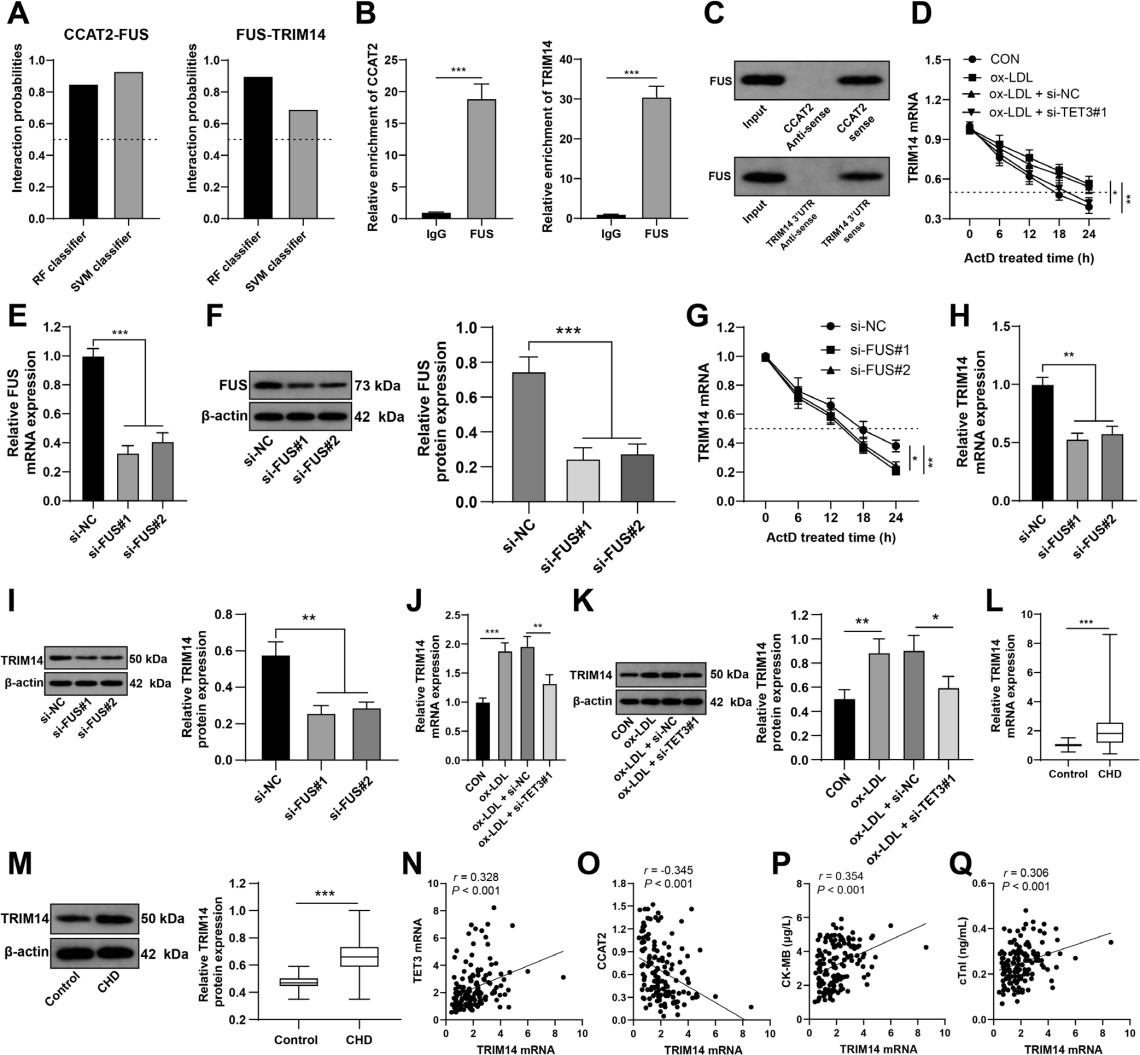
CCAT2 competes with TRIM14 mRNA for binding to FUS, thereby downregulating TRIM14. (A) The possibility of interaction between FUS and CCAT2 (left) and between FUS and TRIM14 (right) is predicted through the RPISeq website. (B) CMEC lysis solution was collected, and a RIP assay was performed using a FUS antibody, and the enrichment of CCAT2 (left) and TRIM14 (right) in the FUS group was compared with that in the IgG group. (C) The binding relationships between CCAT2 and FUS (left) and between TRIM14 and FUS (right) were verified by an RNA pull‐down assay. (D) CMECs transfected with NC or si‐TET3 were exposed to ACT‐D (5 μg/mL), the cellular RNA was separated at the designated time points, and the effect of si‐TET3 on the TRIM14 mRNA half‐life was evaluated by RT–qPCR. (E and F) FUS mRNA and protein expression in CMECs with si‐TET3 was verified by RT–qPCR (E) and western blot analysis (F). (G) CMECs were transfected with CMECs and then treated with ACT‐D to block endogenous transcription to assess TRIM14 mRNA expression at designated time points using RT–qPCR. (H and I) TRIM14 mRNA and protein expression in CMECs with si‐TET3 was detected by RT–qPCR (H) and western blot analysis (I). (J and K) TRIM14 mRNA and protein expression in CMECs transfected with si‐FUS was determined by RT–qPCR (J) and western blot analysis (K). (L and M) TRIM14 mRNA and protein expression in the healthy control group (*n* = 120) and the ACS group (*n* = 142). (N–Q) Relationships of TRIM14 mRNA expression in ACS patients with TET3 mRNA (N), CCAT2 (O), and CK‐MB (P) cTnI (Q) levels were analyzed by Pearson's correlation coefficient. Three independent experiments were performed. The data are presented as the mean ± standard deviation. A *t* test was used to compare the data between two groups in Panels B, L and M; one‐way ANOVA was used to compare the data among multiple groups in Panels E, F, and H–K; and two‐way ANOVA was used to compare the data among multiple groups in Panels D and G. Tukey's multiple comparisons test was used for post hoc tests. *p < 0.05, **p < 0.01, ***p < 0.001.

To elucidate the possible crosstalk of CCAT2 in CMEC injury, the correlation between CCAT2 and FUS was predicted through the RPISeq website. The scores of the RF classifier and SVM classifier were 0.85 and 0.93, respectively (Figure 4A), indicating that FUS might bind to CCAT2. TRIM14 accelerated ox‐LDL‐induced CMEC injury [14], while the RPISeq website revealed the interaction between FUS and TRIM14 (Figure 4A). RIP assays revealed that FUS could bind to CCAT2 and TRIM14 mRNA in CMECs (both *p* < 0.001; Figure 4B). RNA pull‐down assays revealed that CCAT2 and TRIM14 mRNA could both directly bind to FUS (Figure 4C). ACT‐D treatment resulted in an elongated half‐life of TRIM14 mRNA in the ox‐LDL group but a shortened half‐life of TRIM14 mRNA in the si‐TET3 group (all *p* < 0.001; Figure 4D). CMECs were transfected with si‐FUS (*p* < 0.001; Figure 4E,F) and treated with ACT‐D, leading to a shortened TRIM14 mRNA half‐life (*p* < 0.05; Figure 4G). RT–qPCR and the western blot analysis revealed that in CMECs, si‐FUS resulted in downregulation of TRIM14 (*p* < 0.05; Figure 4H,I), whereas ox‐LDL increased TRIM14 expression and si‐TET3 inhibited TRIM14 expression (*p* < 0.05; Figure 4J,K). Compared with the control group, the ACS group presented upregulated TRIM14 (*p* < 0.001; Figure 4L,M). Pearson's correlation coefficient revealed that TRIM14 mRNA expression in serum from ACS patients was positively related to the levels of TET3 mRNA, CK‐MB, and cTnI (all *r* > 0, *p* < 0.001) and was negatively linked to CCAT2 expression (*r* < 0, *p* < 0.001) (Figure 4N–Q). Overall, CCAT2 competes with TRIM14 mRNA for binding to FUS, thereby downregulating TRIM14.

### 3.5 CCAT2 Knockdown Partially Reverses the Protective Role of Si‐TET3 in CMEC Damage

**FIGURE 5:**
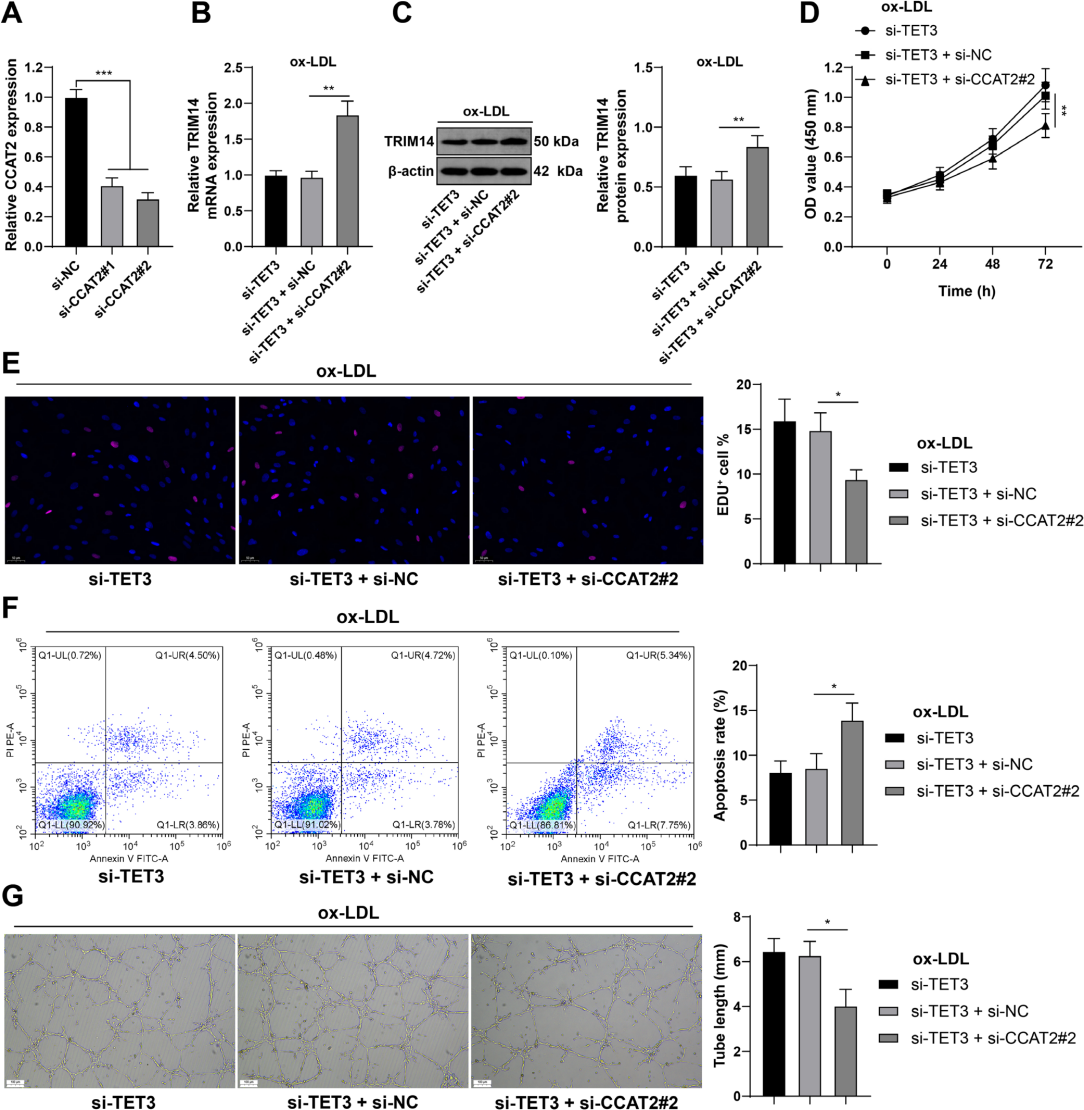
CCAT2 knockdown partially reverses the protective effect of si‐TET3 on CMEC damage. (A) CCAT2 expression in CMECs with si‐CCAT2 was detected by RT–qPCR. (B and C) TRIM14 mRNA and protein expression in CMECs was determined by RT–qPCR (B) and western blot analysis (C). (D) CMEC proliferation was verified by the CCK‐8 method. (E) DNA repair ability of CMECs was evaluated by an EdU assay. (F) CMEC apoptosis was evaluated by flow cytometry. (G) The ability of CMECs to form vascular rings in vitro was verified by a matrix tube formation assay. Three independent experiments were performed. The data are presented as the mean ± standard deviation. One‐way ANOVA was used to compare the data among multiple groups in Panels A–C, E–G, and two‐way ANOVA was used to compare the data among multiple groups in Panel D. Tukey's multiple comparisons test was used for post hoc tests. *p < 0.05, **p < 0.01, ***p < 0.001.

CCAT2 was downregulated in CMECs when si‐CCAT2#2 was used, which resulted in better knockdown efficiency (*p* < 0.001; Figure 5A). RT–qPCR and the western blot analysis revealed that CCAT2 knockdown increased TRIM14 mRNA and protein expression in CMECs (all *p* < 0.01; Figure 5B,C). The results of functional assays revealed that CCAT2 knockdown counteracted the effects of TET3 silencing on CMEC proliferation (*p* < 0.05; Figure 5D), apoptosis (*p* < 0.05; Figure 5E), and angiogenesis (*p* < 0.05; Figure 5F), suggesting that CCAT2 knockdown partially reversed the protective effect of si‐TET3 on CMEC damage.

### 3.6 TRIM14 Overexpression Partially Reverses the Protective Effect of Si‐TET3 on CMEC Damage

**FIGURE 6:**
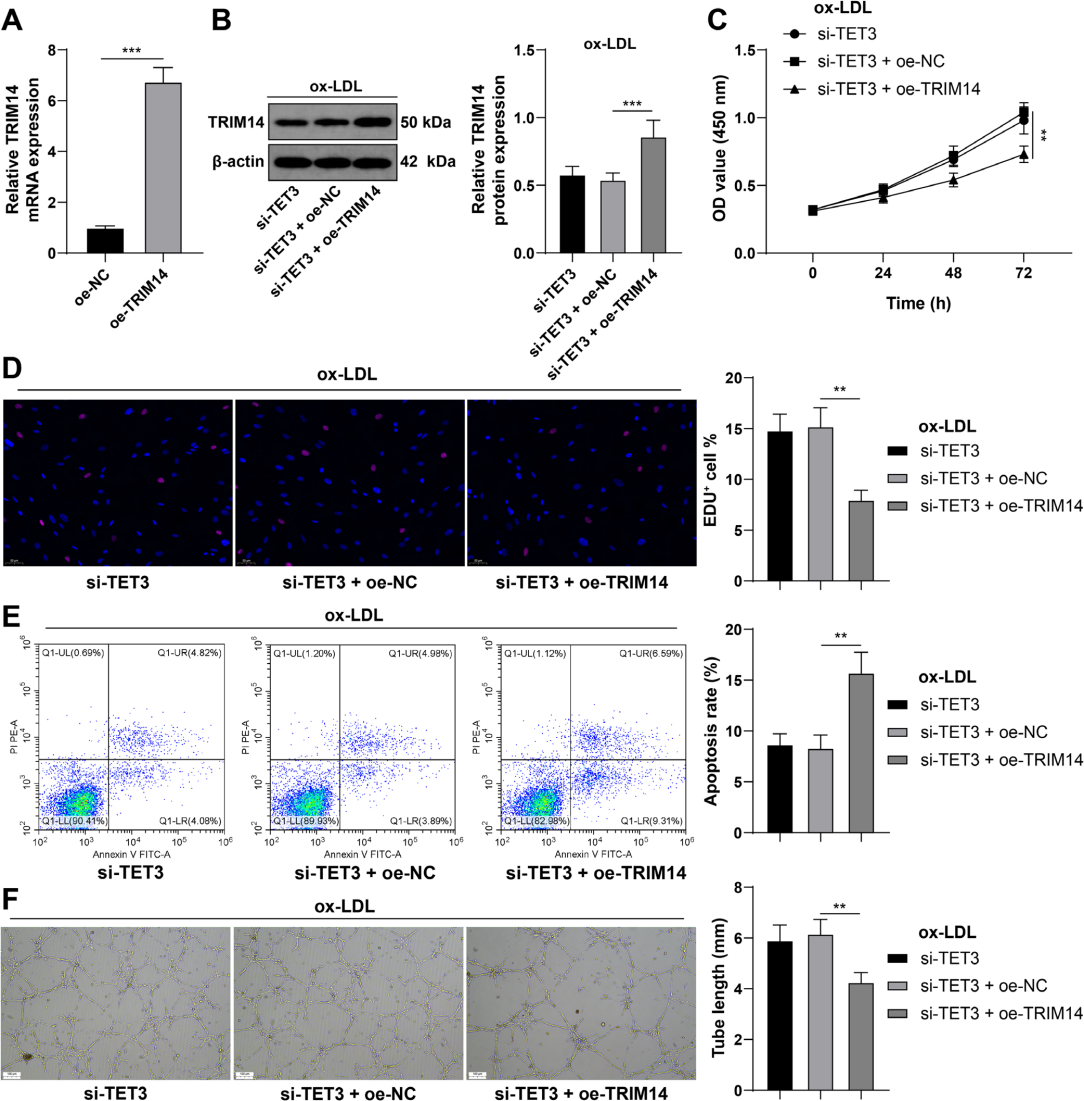
TRIM14 partially reverses the protective effect of si‐TET3 on CMEC damage. (A) TRIM14 mRNA expression in CMECs with oe‐TRIM14 was detected by RT–qPCR. (B) FUS and TRIM14 mRNA and protein expression in CMECs was determined by western blot analysis. (C) CMEC proliferation was assessed by the CCK‐8 method. (D) DNA replication of CMECs was measured by an EdU assay. (E) CMEC apoptosis was evaluated by flow cytometry. (F) The ability of CMECs to form vascular rings in vitro was verified by a matrix tube formation assay. Three independent experiments were performed. The data are presented as the mean ± standard deviation. One‐way ANOVA was used to compare the data among multiple groups in Panels A, D, E, and F, and two‐way ANOVA was used to compare the data among multiple groups in Panels B and C. Tukey's multiple comparisons test was used for post hoc tests. **p < 0.01, ***p < 0.001.

Combined experiments revealed that TRIM14 was upregulated in CMECs (*p* < 0.001; Figure 6A), whereas TET3 expression was silenced (*p* < 0.001; Figure 6B). The results of functional assays revealed that TRIM14 overexpression counteracted the effects of TET3 silencing on CMEC proliferation (*p* < 0.05; Figure 6C), apoptosis (*p* < 0.05; Figure 6D), and angiogenesis (*p* < 0.05; Figure 6E), suggesting that TRIM14 overexpression partially reversed the beneficial effect of si‐TET3 on CMEC damage.

## 4 Discussion

**FIGURE 7:**
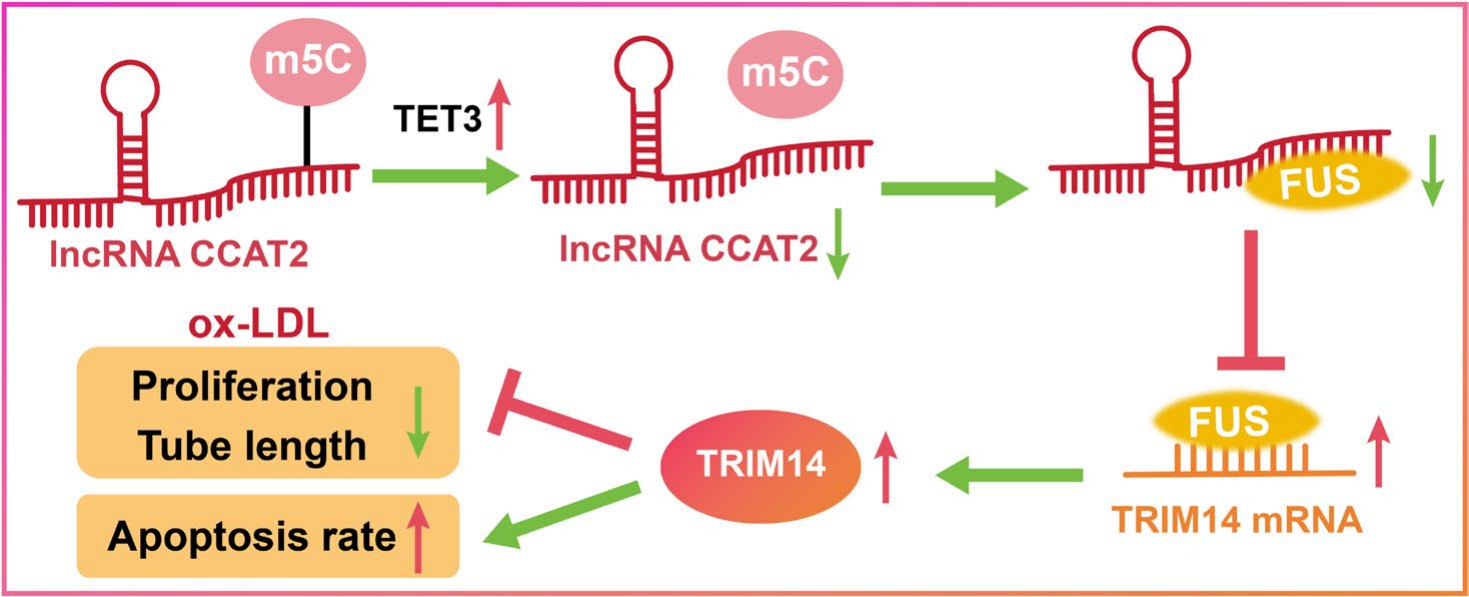
TET3 removes m5C modification to inhibit CCAT2 expression, reduces the binding relationship between CCAT2 and FUS, and promotes the binding relation between FUS and TRIM14 mRNA to upregulate TRIM14, thereby enhancing CMEC damage in ACS.

Despite the development of methods for monitoring and managing CHD, the complex symptoms and prognostic outcomes pose challenges in CHD treatment, and CMEC damage and deficiency are emerging problems demanding prompt solutions [1]. ox‐LDL can simulate the microenvironment of ACS by activating endothelial cells, inducing oxidative stress, promoting inflammatory responses, and triggering thrombosis, and ox‐LDL‐induced microvascular endothelial injury is widely used to mimic the pathological process of ACS [20, 21]. As a class of iron‐dependent dioxygenases, TETs significantly increase m5C modification and DNA methylation on different genes to alter cell biological activities, inflammatory cytokine release, and hematopoietic function under pathological conditions [22]. TET3 stimulates inflammatory reactions, disrupts cardiac monocyte growth, triggers myocardial remodeling, and increases myocardial infarction injury [23], indicating that TET3 might be an adverse factor in the treatment of heart diseases. Our study was designed to explore possible treatments for CHD involving TET3 and its downstream mechanism. Ultimately, we found that TET3 removes m5C modification to inhibit CCAT2 expression, reduces the binding relationship between CCAT2 and FUS, and promotes the binding relation between FUS and TRIM14 mRNA to upregulate TRIM14, thereby enhancing CMEC damage in CHD (Figure 7).

Recent studies have revealed that TET3 function is context dependent, with its effects varying across cell types and pathological states. For instance, TET3 enhances neuroprotection by demethylating BDNF and attenuating neuroinflammation and depression‐like behaviors in mice [24]. TET3 promotes HR‐mediated DNA repair to alleviate cardiac fibrosis [25]. Additionally, TET3 regulates the HIF‐1α/VEGF signaling pathway to accelerate tumor angiogenesis and cell proliferation in thyroid cancer [26]. Moreover, TET3 upregulates IL‐6, contributing to the accelerated progression of atherosclerosis [27]. The most significant finding in this study was that TET3 was strongly expressed in serum from CHD patients and ox‐LDL‐stimulated CMECs and that silencing TET3 could alleviate ox‐LDL‐induced CMEC damage by promoting CMEC proliferation, preventing apoptosis, and increasing angiogenesis. TET3 expression is elevated in the hearts of high‐fat‐diet‐treated mice, which is linked to increased oxidative injury, cardiac failure, heart remodeling, and cardiomegaly in cardiovascular disorders [28]. TET3 impairs vascular smooth muscle cell growth and accelerates apoptosis progression to exacerbate heart dysfunction in patients with CAD [7]. Similarly, when TET3 expression is inhibited in vascular diseases, primary venous EC development is improved, angiogenesis is enhanced, and drug efficacy is strengthened [29]. Overall, TET3 has been reported to be a dangerous indicator in ACS. The increase in TET3 levels may reflect an acute ischemic stress response, which is fundamentally distinct from the chronic pathophysiological processes observed in patients with stable CHD. Further validation in populations with stable CHD is warranted to clarify this potential mechanistic difference. ROC curve analysis revealed that TET3 has considerable diagnostic value for ACS. Therefore, the downstream mechanism of TET3 was further investigated.

Our data supported that TET3 removed the m5C modification on CCAT2 to reduce its stability and expression. Functionally, TET3 interferes with multiple biological pathways by influencing m5C modification, promoting the conversion of m5C to 5hmC, modulating DNA transcription, and mediating cellular activity [30]. The transformation of m5C to 5hmC is pivotal for the construction of DNA fragments and the modulation of gene demethylation and molecular alterations under different pathological conditions [31]. TET3 induces m5C modification on lncRNAs to exacerbate trophoblast dysfunction, retard artery remodeling in preeclampsia, and trigger complications [32]. Abnormally expressed lncRNAs are crucial biological targets of CAD pathogenesis and prevention because they influence angiogenesis, coronary artery occlusion, and EC damage [33]. DNA modification can regulate CCAT2 expression to affect tumor progression [34], but the role of DNA modification on CCAT2 in non‐malignant events remains unexplored, let alone the potential crosstalk of CCAT2. CCAT2 reversed heart function and minimized cardiomyocyte damage and death through competitive binding to BMI1 with miR‐539‐3p [9]. Different lncRNAs and mRNAs are actively involved in CAD, and the interaction between them could expand the understanding of CAD progression and treatment [35]. Herein, the possible mechanism of CCAT2 in CHD was explored through in‐depth research.

CCAT2 competitively bound to FUS with TRIM14 mRNA to downregulate TRIM14. FUS binds to different lncRNAs to affect DNA repair, metabolic activity, neuronal growth, and disease progression [36]. In addition, FUS binds to TRIM14 and increases its stability and expression [37]. FUS levels are elevated in myocardial infarction, which is associated with cardiomyocyte damage, oxidative stress, cell death, and cardiac failure [38]. These findings further increase our confidence in determining the potential interactions among CCAT2, TRIM14, and FUS in CHD. To elucidate the role of CCAT2 in CHD, it was experimentally downregulated in CMECs, which elevated TRIM14 mRNA and protein expression in CMECs and reduced CMEC proliferation and angiogenesis but activated apoptosis. The effect of TRIM14 on CHD‐induced CMEC damage was assessed, and the results revealed that TRIM14 overexpression reduced CMEC proliferation and angiogenesis while activating apoptosis. Strongly expressed TRIM14 leads to cardiac fibrosis, cardiomyocyte loss, and heart hypertrophy [39]. TRIM14 inhibits CMEC activity and angiogenesis and catalyzes inflammatory reactions, oxidative stress, and CMEC damage [40]. In conclusion, both CCAT2 knockdown and TRIM14 overexpression partially reversed the protective effect of si‐TET3 on CMEC damage.

Collectively, our findings indicated that TET3 expression was activated in serum from CHD patients and ox‐LDL‐treated CMECs. TET3 removed m5C modification to inhibit CCAT2 expression and reduced the binding relationship between CCAT2 and FUS to upregulate TRIM14, thereby enhancing CMEC damage in CHD. We are the first to demonstrate that TET3 regulates TRIM14 expression via RNA m5C epigenetic modification. Furthermore, we elucidated the precise mechanism underlying the dynamic regulation of TRIM14 by RNA m5C epigenetic modifications in the microvascular endothelium of human CHD, thereby identifying a novel regulatory pathway for therapeutic intervention. This study has several limitations. First, we did not conduct mass spectrometry to screen for m5C modifications on other possible RNAs regulated by TET3 because of the limited experimental funding. In addition, owing to funding constraints, this study mainly explored the mechanism by which TET3 mediates the m5C modification of the lncRNA CCAT2 in microvascular endothelial cell injury through in vitro CMEC experiments, and more in vivo studies are needed to elucidate the specific mechanism of TET3 in CHD. The causal role of FUS in mediating the effect of TRIM14 on endothelial injury will be addressed in an independent study focusing on FUS signal transduction. The sample size needs to be expanded to enhance the efficacy of clinical validation. We will continue to elucidate the role of TET3 in m5C modification on other RNAs in CHD and the upstream mechanism of TET3 to provide a new theoretical foundation for CHD treatment.

## Funding

This work was supported by National Center for Cardiovascular Diseases, Central China Branch Self‐contained Projects (2023‐FZX13) and Henan Province Young and Middle‐Aged Health Science and Technology Innovation Talent Project (LJRC2023011).

## Conflicts of Interest

The authors declare no conflicts of interest.

## Data Availability

The data that support the findings of this study are available from the corresponding author upon reasonable request.
